# Dysbiosis and Depletion of Fecal Organic Acids Correlate With the Severity of Rejection After Rat Liver Transplantation

**DOI:** 10.3389/ti.2022.10728

**Published:** 2022-09-15

**Authors:** Siyuan Yao, Shintaro Yagi, Eri Ogawa, Masaaki Hirata, Yosuke Miyachi, Sena Iwamura, Ryuji Uozumi, Takuya Sugimoto, Takashi Asahara, Shinji Uemoto, Etsuro Hatano

**Affiliations:** ^1^ Department of Surgery, Graduate School of Medicine, Kyoto University, Kyoto, Japan; ^2^ Department of Surgery, Graduate School of Medicine, Kanazawa University, Ishikawa, Japan; ^3^ Department of Biomedical Statistics and Bioinformatics, Graduate School of Medicine, Kyoto University, Kyoto, Japan; ^4^ Yakult Central Institute, Yakult Honsha Co., Ltd., Tokyo, Japan; ^5^ Shiga University of Medical Science, Otsu, Japan

**Keywords:** T cell-mediated rejection, dysbiosis, predominant obligate anaerobes, ribosomal RNA-targeted reverse-transcription quantitative polymerase chain reaction, short-chain fatty acid

## Abstract

The impact of T cell-mediated rejection (TCMR) after liver transplantation (LT) on the alterations in the gut microbiota (GM) and associated intestinal environment represented by fecal organic acids (OAs) require further elucidation. A rat allogeneic LT model was prepared without immunosuppressants or antibiotics, and a syngeneic model was used as a control. Qualitative and quantitative analyses of fecal samples at fixed time points were performed. Correlation analyses were also performed between liver function and GMs and OA levels. In the allogeneic TCMR group, the number of predominant obligate anaerobes decreased as liver function declined. *Clostridioides difficile*, *Enterobacteriaceae*, *Enterococcus*, *Streptococcus*, and *Staphylococcus* were significantly increased. Regarding fecal OA concentration, short-chain fatty acid (SCFA) concentrations were depleted as liver function declined. In contrast, in the syngeneic group, GM and OAs exhibited only slight, transient, and reversible disturbances. In addition, alanine aminotransferase and total bilirubin were positively correlated with the number of *Enterobacteriaceae* and *Enterococcus,* and negatively correlated with the fecal concentration of SCFAs. The allogeneic TCMR model demonstrated distinct dysbiosis and depletion of fecal OAs as TCMR progressed after LT. The degree of graft injury was closely related to the number of specific bacterial strains and the concentrations of fecal SCFAs.

## GRAPHICAL ABSTRACT

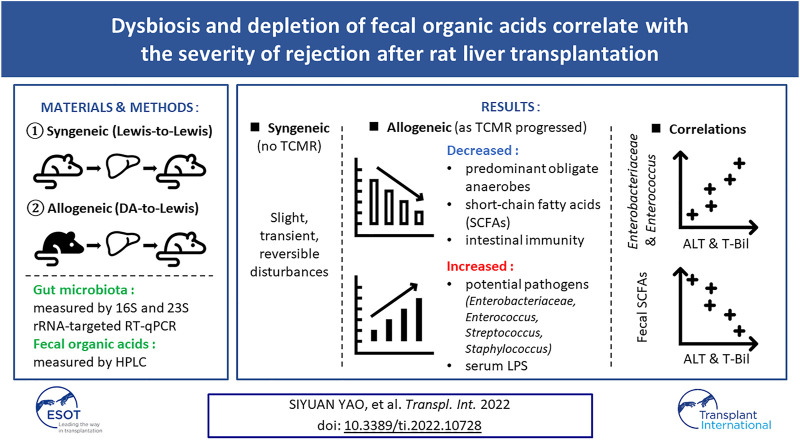

## Introduction

It is well recognized that the gut microbiota (GM) plays an important role in the development of complications of end-stage liver disease (ESLD) including bacterial infections and hepatic encephalopathy (1,2,3), and knowledge has gradually accumulated with regard to the GM composition in liver transplantation (LT) candidates (4,5,6,7). However, accurate interpretation of human GM and the associated intestinal environment, particularly in the peri-LT period, is difficult because they are influenced by miscellaneous factors including surgical stress, perioperative fasting, immunosuppressant use, and antibiotic administration. Therefore, animal experiments that exclude such confounders are required to understand the true traits of the GM.

T cell-mediated rejection (TCMR) is common early after LT. Although mild TCMR does not adversely affect the clinical course when adequately treated, severe TCMR still carries deleterious effects with an associated risk of graft loss and decreased survival (8). Since the target organ of TCMR in LT is the liver, severe TCMR, like other ESLDs, could cause secondary structural and functional changes in the intestine. Although previous experimental studies demonstrated that TCMR induced a structural shift of the GM in rats (9, 10), the clinical impact of graft function on specific strains, and vice versa, has never been investigated. Therefore, existing evidence needs to be updated using the latest technology. Herein, we introduce the 16S and 23S ribosomal RNA (rRNA)-targeted reverse-transcription quantitative polymerase chain reaction (RT–qPCR) system for the detection of microorganisms, which enables more sensitive qualitative and quantitative analyses than conventional real-time qPCR.

Advances in technology have made it possible to visualize the intestinal environment by evaluating not only GM but also fecal organic acids (OAs). Fecal OAs, especially short-chain fatty acids (SCFAs), including acetic acid, butyric acid, and propionic acid, produced by the GM are known to have beneficial physiological effects on host immunity through the suppression of the overgrowth of harmful microorganisms (11), protection of the intestinal epithelium (12), and regulation of intestinal immune function (13). Therefore, SCFAs would have a direct and decisive effect on maintaining host immunity and minimizing bacterial translocation (BT) (14, 15). Chronological changes in OA as a decisive consequence of GM alterations have never been investigated in the TCMR model.

To answer these clinical questions, a rat allogeneic LT model was prepared without fasting, antibiotic treatment, or immunosuppressant administration to monitor perioperative time-series changes in both GM and fecal OA levels complicated by impaired liver function caused by TCMR. A rat syngeneic LT model, which showed different transitions during liver function recovery, was concurrently prepared as a control. The goals of the current study were 3-fold:

(1) To observe the dynamic alterations of both the GM and fecal concentrations of OAs in the syngeneic and allogeneic LT model.
(2) To elucidate the interactions between graft liver function and these two variables (the GM and fecal OAs).
(3) To better assess the causal relationship between TCMR and BT.

## Materials and Methods

### Experimental Protocol

Male Lewis rats (9–12 weeks old) weighing 270–320 g and male Dark Agouti (DA) rats (12–16 weeks old) weighing 260–290 g were prepared. The whole liver graft was transplanted after 1 h of cold storage in phosphate-buffered saline. The median weight of grafts from Lewis and Dark Agouti rats was 10.465 g (range, 9.250–11.600) and 8.001 g (range, 7.510–8.888), respectively. Rats were divided into two groups after orthotopic liver transplantation (OLT): 1) the syngeneic group (*n* = 6), in which both the donors and recipients were Lewis rats; and 2) the allogeneic group (*n* = 6), in which the donors were DA rats and the recipients were Lewis rats. Fecal, blood, and histological (liver and small intestine) samples were obtained at four fixed time points (days 1, 3, 7, and 10) after OLT. In total, 48 OLTs were performed for 24 individuals in each group (6 individuals × 4 time points). Six healthy Lewis rats were used as the controls. As the present basic research is an exploratory study, a power calculation was not performed. We selected this relatively small sample size empirically because the GM and fecal OAs were measured simultaneously for rats for the first time, and therefore, the initial intention was to gather basic evidence regarding the transitions of these variables that could be utilized in future human studies. All OLT procedures were performed under inhalation anesthesia using 1.5% isoflurane with endotracheal intubation and artificial respiration, according to our previous techniques, with hepatic artery reconstruction (16) and without fasting, intravenous drip, antibiotic administration, or immunosuppression. Animals were housed under specific pathogen-free conditions in a temperature- and humidity-controlled environment under a 12-h light/dark cycle. The rats were fed a standard diet (F-2; Oriental Bio Service, Kyoto, Japan) and tap water *ad libitum*.

All experiments were conducted in accordance with the Animal Research: Reporting of *In Vivo* Experiments (ARRIVE) Guidelines. The institutional ethics committee of Kyoto University approved the experimental protocol (MedKyo18537).

### Sample Collection

Under inhalation anesthesia, portal venous pressure (PVP) was measured and monitored *via* a pressure transducer using the following procedure: a segment of the mesenteric branch vein was cannulated with a 24-g cannula needle, and the tip of the cannula was advanced into the trunk of the superior mesenteric vein. Blood samples were collected from the inferior vena cava and feces from the rectum. Each individual was euthanized at each time point after sample collection.

The fecal samples were placed directly into two tubes (∼1.0 g/tube); one tube contained 2 ml of RNAlater® (Ambion, Austin, TX, United States), and the other was empty. The samples with RNAlater® were held at room temperature for 10 min before storage at 4°C (for the analysis of GM), and the others were placed in a freezer at −80°C (for the analysis of fecal OA concentrations) within 30 min of excretion. Samples were sent to the Yakult Central Institute at −20°C for analysis.

### Determination of Fecal Microbiota Counts

GM composition was analyzed by the 16S and 23S rRNA-targeted RT–qPCR system using Yakult Intestinal Flora-SCAN (YIF-SCAN®). The mechanisms and advantages of YIF-SCAN® for measuring bacterial counts in fecal and blood samples have been previously described elsewhere (17,18,19). Briefly, three serial dilutions of the extracted RNA sample were used for bacterial rRNA-targeted RT-qPCR, and threshold cycle values in the linear range of the assay were applied to the standard curve to obtain the corresponding bacterial cell count in each nucleic acid sample. These data were then used to calculate bacterial counts per sample. The specificity of the RT-qPCR assay using group-, genus-, or species-specific primers was determined as previously described (Supplementary Table S1).

The bacteria examined included obligate anaerobes (*Clostridium coccoides* group, *C. leptum* subgroup, *Bacteroides fragilis* group, genus *Bifidobacterium*, *Atopobium* cluster, genus *Prevotella*, *Clostridioides difficile*, and *C. perfringens*), facultative anaerobes (family *Enterobacteriaceae*, genus *Enterococcus*, genus *Streptococcus*, and genus *Staphylococcus*), and aerobes (genus *Pseudomonas*).

### Determination of Fecal OA Concentrations

A portion of the feces was homogenized in four volumes of 0.15 mol/L perchloric acid and stored at 4°C for 12 h. The homogenate was centrifuged at 4°C at 20,400 ×g for 10 min, and the resulting supernatant was passed through a membrane filter with a pore size of 0.45-μm (Millipore Japan Ltd., Tokyo, Japan). The sample was analyzed by high-performance liquid chromatography using a Waters system with Waters 432 Conductivity Detector (Waters Co., Milford, MA) equipped with two columns (Shodex RS pack KC-811; Showa Denko Co. Ltd., Tokyo, Japan).

In this study, the SCFAs included acetic acid, butyric acid, and propionic acid.

### Biochemical Assays

Blood tests, including complete blood count, peripheral neutrophil/lymphocyte ratio (NLR), aspartate aminotransferase, alanine aminotransferase (ALT), total bilirubin (T-Bil), serum albumin, and serum creatinine, were performed in a professional clinical laboratory (Japan Clinical Laboratories, Kyoto, Japan).

To evaluate the immune function, the CD4/CD8 T-cell ratio was analyzed. The conjugated mouse anti-rat monoclonal antibodies used for flow cytometry, APC-conjugated CD3, FITC-conjugated CD4, and PE-conjugated CD8a, were commercially available (BD Biosciences, San Josè, CA, United States). Samples were acquired using a BD Accuri C6 (BD Biosciences).

The fecal IgA content was determined to evaluate intestinal barrier function by enzyme-linked immunosorbent assay (ELISA) using a rat IgA ELISA kit (Bethyl Laboratories, Inc., Montgomery, TX, United States). Serum lipopolysaccharide (LPS) levels were evaluated using a rat LPS ELISA kit (CUSABIO, Wuhan, China).

### Histological Analysis

Formalin-fixed, paraffin-embedded sections (4-μm thickness) of rat liver grafts and small intestines were stained with hematoxylin and eosin (H&E). For electron microscopy, rat small intestines were perfused through the aorta with a mixture of 2% glutaraldehyde and 4% paraformaldehyde and then extracted. The intestines were cut into small pieces and stored overnight at 4°C. The sections were stained with saturated uranyl acetate and lead citrate and observed using a Hitachi H-7650 electron microscope (Hitachi, Tokyo, Japan) for transmission electron microscopy (TEM). Two independent investigators examined all the tissue sections in a blinded manner. The severity of TCMR was evaluated in accordance with the Banff classification (20).

### Statistical Analysis

The results of the fecal GM and OA analyses are expressed as the mean ± standard error. For statistical calculation, a value of half of the detection limit was assigned when the count or concentration was below the detection limit. Longitudinal data of these variables were analyzed using a linear mixed-effects model, which included the study group, time after LT, and interaction of the study group with the time after LT. Other continuous variables were presented as the median and range or interquartile range (IQR), as appropriate. Categorical variables were presented as numbers and percentages. Correlations between two variables were determined using Spearman’s rank correlation coefficient. Statistical significance was set at a *p* value < 0.05. JMP 14.0 (SAS Institute, Cary, NC, United States) was used for all statistical analyses.

## Results

### Experimental Characteristics of Rat LT Models

**FIGURE 1:**
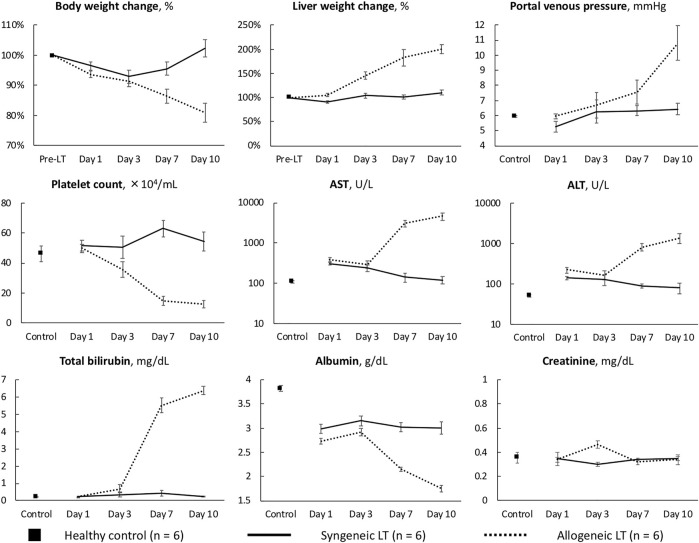
Characteristics of the syngeneic and allogeneic LT models AST, aspartate aminotransferase; ALT, alanine aminotransferase.

**FIGURE 2:**
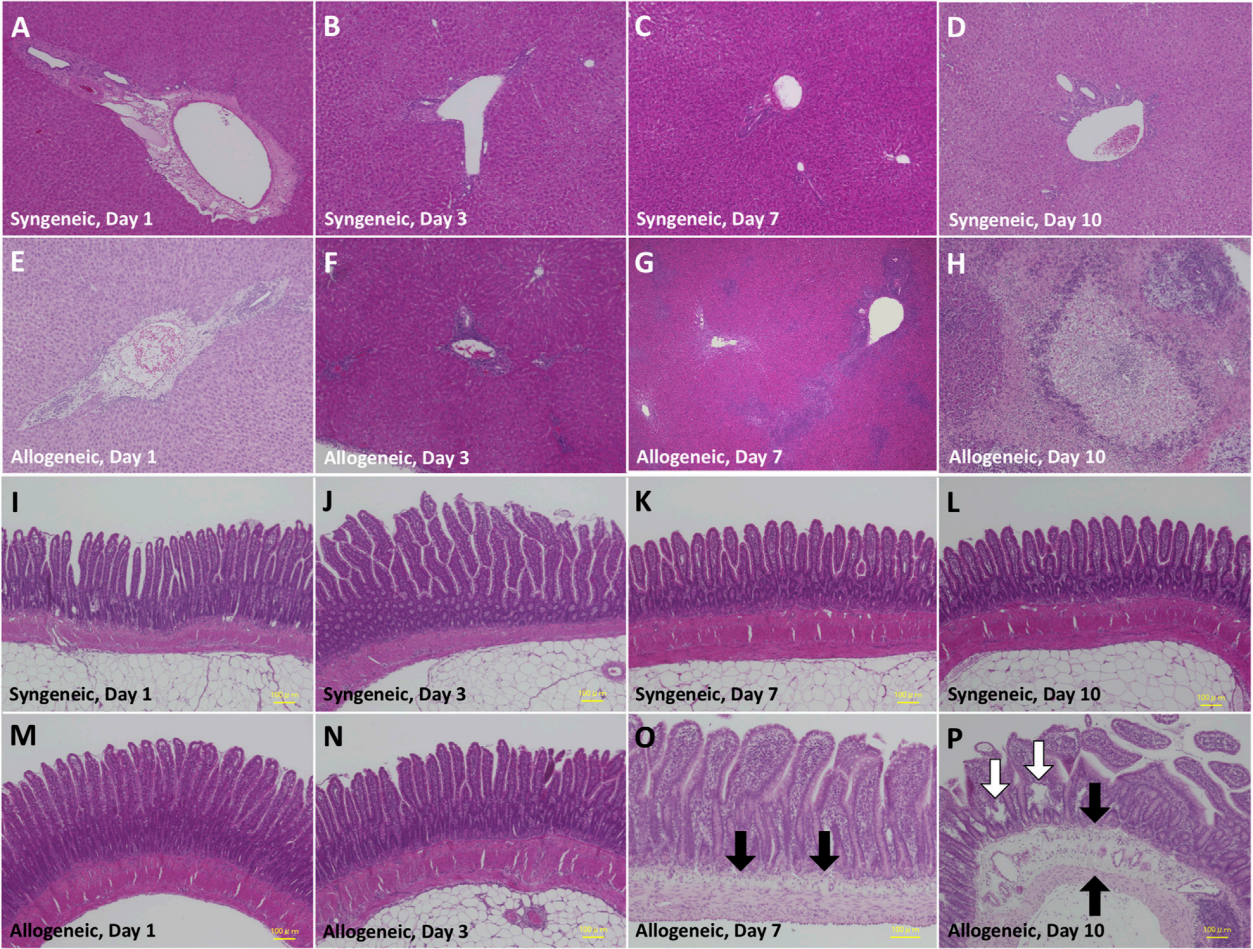
Histopathological alteration of the liver **(A–D)** and small intestine **(E–H)** by hematoxylin-eosin staining. **(A)** Periportal edema due to ischemia/reperfusion injury on day 1. **(B)** Attenuated periportal edema on day 3. **(C)** Almost normal histology on day 7. **(D)** Almost normal histology on day 10. **(E)** Periportal edema due to ischemia/reperfusion on day 1. **(F)** Mild inflammatory cell infiltration in Glisson’s capsule on day 3, with preservation of the structure of the portal vein, hepatic artery, and bile duct.**(G)** Massive inflammatory cell infiltration in Glisson’s capsule and degeneration of hepatocytes in the hepatic lobule on day 7. **(H)** Extensive inflammatory cell infiltration and hepatocyte necrosis on day 10. **(I–N)** Almost normal histology. **(O)** Mild submucosal edema (black arrow) and swollen villi. **(P)** Severe submucosal edema with apparent vascular dilation (black arrow) and disarrangement of the epithelium (white arrow).

**FIGURE 3:**
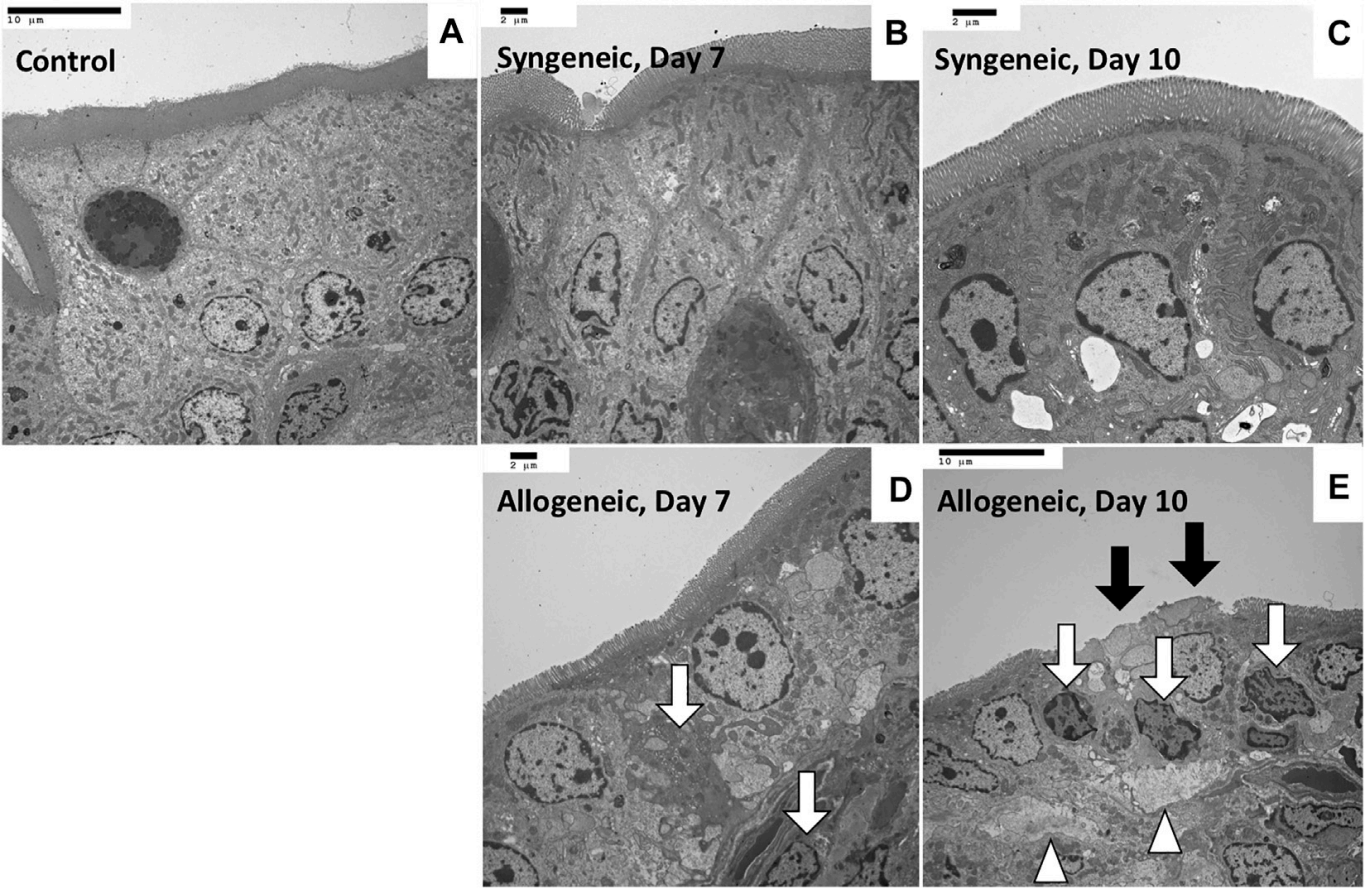
Transmission electron microscopy images of rat small intestine samples **(A)** Control, ×1,200. **(B)** Maintenance of the whole structure, with a clear cell boundary, on day 7, ×1,500. **(C)** Maintenance of the whole structure on day 10, ×2,000. **(D)** Vague boundary of epithelial cells because of partial necrosis (white arrow), ×1,500. **(E)** Microvilli shedding (black arrow), epithelial cell necrosis (arrow), and divergence of intercellular spaces (arrowhead) on day 10, ×1,200.

Representative biochemical analyses (Figure 1) and histopathological findings (Figures 2, 3) are presented. Briefly, after OLT, the hepatic graft suffered from ischemia/reperfusion injury in both syngeneic and allogeneic groups on day 1, with elevated liver enzyme levels (Figure 1) and histological periportal edema (Figures 2A,E). However, the graft recovered to nearly normal levels both functionally and histologically in the syngeneic group (Figures 2B–D), whereas progressive TCMR led to irreversible graft failure after day 7 and by day 10 in the allogeneic group (Figures 2G,H). Figures 2F–H represent “mild,” “moderate,” and “severe” by Banff classification, respectively. As liver enzyme levels increased and cholestasis progressed, synthetic ability decreased, and portal venous pressure increased (Figure 1). Small intestine histology in the allogeneic group showed worsening submucosal edema and disarrangement of the epithelium as TCMR progressed from day 7 to day 10 (Figures 2M–P). Transmission electron microscopy images of the small intestine demonstrated epithelial cell structural destruction as TCMR progressed from day 7, indicating disruption of barrier function (Figure 3).

The median survival period of allogeneic liver grafts was 11 days (range, 10–13), whereas all syngeneic liver grafts survived.

### Time-Series Changes in the GM and OA Concentrations

**FIGURE 4:**
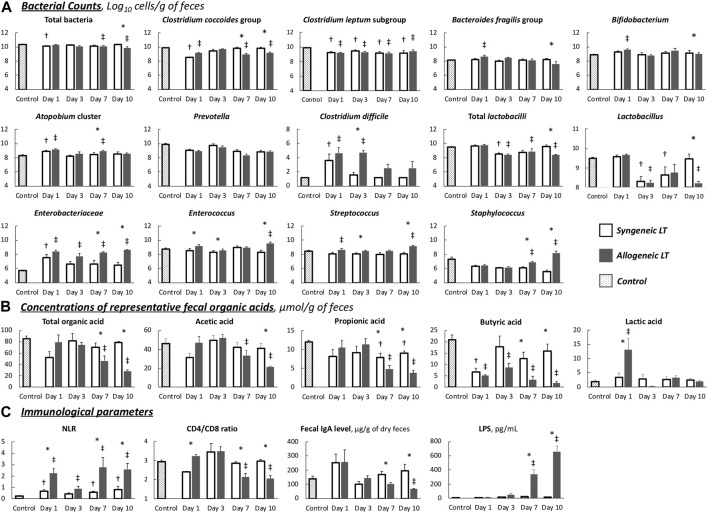
Chronological changes in parameters **(A)** Gut microbiota. **(B)** Organic acids. **(C)** Immunological parameters. Values are the mean (SE), *n* = 6 per group. *p < 0.05, syngeneic group compared to allogeneic group. ^†^ *p* < 0.05, syngeneic group compared to control group. ^‡^ *p* < 0.05, allogeneic group compared to control group. NLR, neutrocyte/lymphocyte ratio; LPS, lipopolysaccharide.

Dynamic comparisons of the representative fecal microbiota are shown in Figure 4A. Dysbiosis progressed as the liver function declined in the allogeneic group, mainly on days 7 and 10, whereas it recovered as the liver function improved in the syngeneic group. The number of predominant obligate anaerobes (POAs), such as the *C. coccoides* group, *B. fragilis* group, and *Bifidobacterium,* decreased as liver function declined in the allogeneic group. These changes were more remarkable in obligate and facultative anaerobes, some of which are responsible for opportunistic infections. Total *lactobacilli* and its subgroup showed a significant decrease, and *Clostridioides difficile*, *Enterobacteriaceae*, *Enterococcus*, *Streptococcus*, and *Staphylococcus* showed a significant increase in the allogeneic group on days 7 and 10, as liver function declined. Meanwhile, GM seemed to be restored to normal by day 10 in the syngeneic group as liver function improved. *C. perfringens*, *Lacticaseibacillus*, and *Pseudomonas* were below the detection limits.

Dynamic comparisons of representative OAs are shown in Figure 4B. Overall, the allogeneic group showed significantly lower OA concentrations as the liver function declined. More specifically, in the allogeneic group, SCFA concentrations were depleted by day 10, with a slight recovery trend from days 1–3. In contrast, the concentration of SCFAs in the syngeneic group recovered to nearly normal levels after depletion on day 1. The remaining values are listed in Supplementary Table S2. The transitions in these values were similar between the groups.

In short, although the disturbance was slight, transient, and reversible in the syngeneic model, the allogeneic TCMR model demonstrated distinct dysbiosis and depletion of fecal OAs.

### Immune Function and Intestinal Barrier Function

In the syngeneic group, all measured values gradually returned to normal by day 10 after LT (Figure 4C). In contrast, immune function, represented by the NLR and CD4/CD8 ratio, and intestinal barrier function, represented by the fecal IgA level, decreased as liver function declined in the allogeneic group. On days 7 and 10, the allogeneic group showed a significantly higher NLR, a lower CD4/CD8 ratio, and decreased fecal IgA levels than the syngeneic group. Consequently, extremely high LPS levels were observed from days 7–10, implying the occurrence of BT.

### Correlations Between Liver Function and the GM and OA Concentrations

**FIGURE 5:**
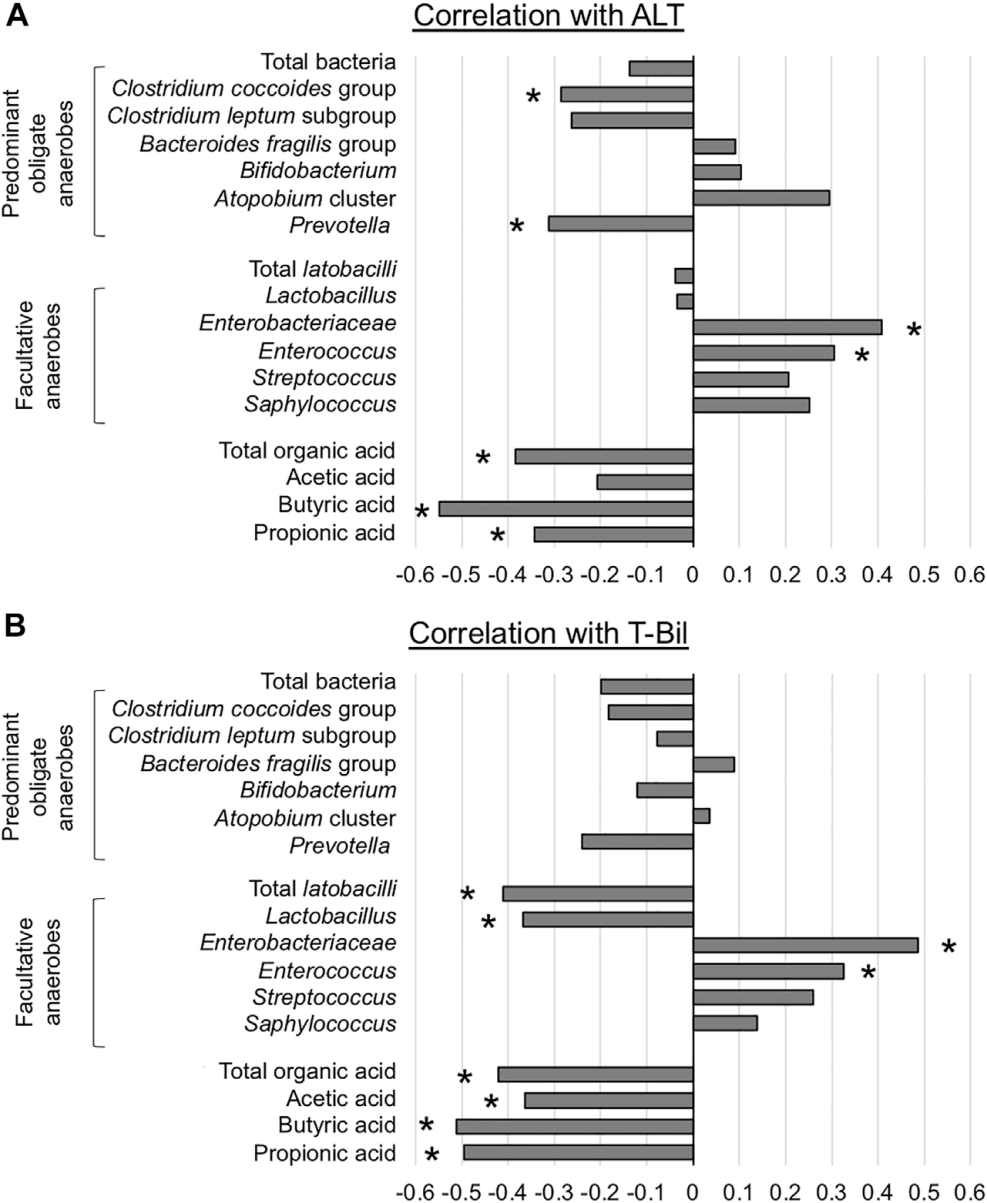
Association between liver function and representative microorganisms and fecal organic acids **(A)** Alanine aminotransferase (ALT) **(B)** Total bilirubin (T-Bil) *p < 0.05, Spearman’s rank correlation coefficient.

Spearman’s rank correlation coefficient was calculated using the data of all 48 OLTs because no single individual was sampled multiple times. ALT was significantly negatively correlated with *C. coccoides* group and *Prevotella* and positively correlated with *Enterobacteriaceae* and *Enterococcus* (Figure 5A). With regard to OA, there were significant negative correlations between ALT and fecal concentrations of total OA, butyric acid, and propionic acid. T-Bil was significantly negatively correlated with total *lactobacilli* and *Lactobacillus* and positively correlated with *Enterobacteriaceae* and *Enterococcus* (Figure 5B). With regard to OA, there were significant negative correlations between T-Bil and fecal concentrations of total OA, acetic acid, butyric acid, and propionic acid.

In summary, liver function affected the counts of *Enterobacteriaceae* and *Enterococcus,* and the fecal concentration of SCFAs.

## Discussion

The valuable strength of this experimental study is the new insight that dysbiosis of the GM and depletion of fecal OAs progressed in proportion to deteriorating graft function caused by TCMR in the absence of intervention, including fasting, immunosuppressant, and antibiotic use. We also demonstrated that severe TCMR could become critically complicated by BT due to impaired intestinal barrier function, while the function was maintained in mild to moderate TCMR. Although these data would help us comprehend GM in the context of liver disease, several issues require discussion.

TCMR is known to induce a structural shift of the GM in rats (9, 10), possibly as a consequence of cholestasis caused by bile duct injury characterizing TCMR (21). A previous study using conventional qPCR showed that *Faecalibacterium prausnitzii* and *Lactobacillus* were significantly reduced in a rat TCMR model with enrichment of *Clostridium bolteae* (10), these microorganisms were not the key pathogens relevant to the clinical practice of LT cited in past studies (7, 22). Our results demonstrated significant alterations in more strains. The number of POAs, such as *C. coccoides* group, *B. fragilis* group, and *Bifidobacterium,* decreased as liver function declined. The changes were more remarkable in obligate and facultative anaerobes: total *lactobacilli* and its subgroup showed a significant decrease, and *C. difficile, Enterobacteriaceae*, *Enterococcus*, *Streptococcus*, and *Staphylococcus* showed a significant increase. This high testing capability is due to the sensitivity of the YIF-SCAN^®^. YIF-SCAN^®^ targets rRNA molecules that are abundant in bacteria (approximately 10^4^ copies per actively growing cell), and its sensitivity is 100 times higher than that of qPCR assays that target rRNA genes (more than 10 copies/bacterial genome) (23). It can only measure live bacteria that are highly associated with infectivity, inflammation induction, and pathogenicity. In contrast, conventional qPCR targets DNA that remains even in dead bacteria. Therefore, this method was able to capture the dynamic changes in the GM more accurately.

In addition to the disturbance in the GM, depletion of OAs, especially SCFAs, was observed under TCMR. Since most POAs, such as *C. coccoides* group, *Bifidobacterium,* and *Lactobacilli* have been reported to produce SCFAs in the intestine (11,24,25,26), the decrease in the number of POAs and other beneficial bacteria might lead to a decreased fecal concentration of SCFAs, which reflects the condition of intestinal dysbiosis and impaired intestinal barrier function. In such conditions, BT tends to occur, and these events can subsequently lead to bacteremia and postoperative infectious complications (15).

In the clinical setting, differentiating between acute TCMR and infection remains a clinical challenge during the early post-LT period. The two diagnoses can even coexist and lead to unfavorable outcomes. Although the GM and OAs were maintained under mild to moderate TCMR, our data showed an increased risk of BT as TCMR progressed, with worsening dysbiosis, depleting SCFAs, and increasing LPS levels. Since the treatment options for TCMR and infection are diametrically opposed, severe TCMR cases may develop infection and have an irreversible course. These results explain one of the reasons why mild TCMR is treatable, while severe TCMR is refractory to treatment with potent immunosuppressants (8). However, administrating therapeutic antibiotics to all patients with suspicious TCMR is unreasonable because antibiotics can further agitate the gut microbiota. Ideally, if TCMR occurs, we will watch for the onset of BT and consider antibiotic administration referring to other objective test results including inflammatory maker levels and pathology by liver biopsy. Although a comprehensive decision is needed in the clinical setting, speculation can be raised based on our results that if TCMR is pathologically mild to moderate, antibiotics are not necessary, and if it is moderate to severe, they might be required to prevent BT.

According to the correlation analyses, the counts of *Enterobacteriaceae* and *Enterococcus* were positively correlated with ALT and T-Bil*,* and consequently, the fecal concentration of SCFAs was negatively correlated. These interactions suggest that impaired liver function would provoke increases in potential pathogens and intestinal barrier dysfunction, which is an important aspect of the gut-liver axis. In clinical practice, *Enterobacteriaceae* and *Enterococcus* are the dominant pathogens in LT recipients (7, 22). Moreover, *Enterobacteriaceae* and *Enterococcus* have been reported to be enriched in various chronic liver diseases, including viral hepatitis, alcoholic abuse, non-alcoholic steatohepatitis, and cholestatic liver disease (27,28,29,30,31). In addition, considering that the correlation with SCFAs was stronger for T-Bil than for ALT, cholestasis might be the main contributor to the pathogenesis of these phenomena rather than the hepatocellular damage itself. Although bile acids are known to play an essential role in regulating the intestinal immune system (32,33,34), our findings confirmed that SCFAs depletion is a key mechanism connecting dysbiosis caused by reduced amounts of bile acids within the intestine and intestinal barrier dysfunction in liver disease.

This experimental model also provides a clue to comprehend the impact of surgical invasiveness on the intestinal environment. Our results demonstrated that the alterations in both GM and OAs until day 3 were slight, transient, and reversible without TCMR. These changes reflect the true influence of surgical stress because perioperative fasting and antibiotic use, which could impact GM, were not conducted. While surgical procedures are reported to cause large alterations in the GM and OAs in human subjects (35, 36), we assume that it would largely account for concomitant long-term fasting or antibiotic use.

The current study has several limitations. First and foremost, a concurrent allogeneic LT model with immunosuppressants was not prepared. Since many immunosuppressive drugs have been reported to induce dysbiosis in rodent models (37), we intended to exclude the potential confounder in interpreting the GM. Besides, our results demonstrated that the GM and OAs strongly correlate with graft function; thus, the fluctuations would be minimal as long as graft function is maintained with the aid of immunosuppressants. Second, we could not provide data on the underlying mechanisms of the altered intestinal environment. More specific analysis based on individual strains and their metabolites is demanding. Finally, experimental findings implicating individual organisms or genera in animal models are less valuable until they are validated in humans. Although our next step is to investigate perioperative changes in GM in human LT recipients, it is expected that miscellaneous confounders would make interpretation difficult in human subjects. Hopefully, the findings of this experimental study will provide clues for interpreting the results of future research.

In conclusion, distinct characteristics of both GM and fecal concentrations of OAs in the TCMR model were visualized. While successful LT would have little influence on the GM and intestinal environment, TCMR could increase pathogenic strains, weaken intestinal immune function, and elevate the potential risk of BT. In addition, the degree of graft injury is closely related to the counts of some specific bacterial strains and the concentrations of fecal SCFAs; thus, rejection and infection may coexist when rejection is uncontrollable.

## Data Availability

The raw data supporting the conclusion of this article will be made available by the authors, without undue reservation.

## Abbreviations

ALT, alanine aminotransferase; BT, bacterial translocation; ELISA, enzyme-linked immunosorbent assay; ESLD, end-stage liver disease; GM, gut microbiota; IQR, interquartile range; LDLT, living donor liver transplantation; LPS, lipopolysaccharide; LT, liver transplantation; NLR, neutrophil/lymphocyte ratio; OA, organic acid; OLT, orthotopic liver transplantation; POAs, predominant obligate anaerobes; RT-qPCR, reverse-transcription quantitative polymerase chain reaction; SCFA, short-chain fatty acid; T-Bil, total bilirubin; TCMR, T cell-mediated rejection.
