# Supplementary material for: Dysbiosis and Depletion of Fecal Organic Acids Correlate With the Severity of Rejection After Rat Liver Transplantation
Source: Transpl Int. 2022 Sep 15;35:10728. doi: 10.3389/ti.2022.10728 (PMC9519788; doi:10.3389/ti.2022.10728)
Supplement: Supplementary file 1 [file DataSheet1.docx]

**Table S1**

| Target bacteria | Primer | Sequence (5’ - 3’) |
| --- | --- | --- |
| *Clostridium coccoides* group | g-Ccoc-F | AAATGACGGTACCTGACTAA |
|  | g-Ccoc-R | CTTTGAGTTTCATTCTTGCGAA |
| *Clostridium leptum* subgroup | sg-Clept-F | GCACAAGCAGTGGAGT |
|  | sg-Clept-R3 | CTTCCTCCGTTTTGTCAA |
| *Bacteroides fragilis* group | g-Bfra-F2 | AYAGCCTTTCGAAAGRAAGAT |
|  | g-Bfra-R | CCAGTATCAACTGCAATTTTA |
| *Bifidobacterium* | g-Bifid-F | CTCCTGGAAACGGGTGG |
|  | g-Bifid-R | GGTGTTCTTCCCGATATCTACA |
| *Atopobium* cluster | g-Atopo-F | GGGTTGAGAGACCGACC |
|  | g-Atopo-R | CGGRGCTTCTTCTGCAGG |
| *Prevotella* | g-Prevo-F | CACRGTAAACGATGGATGCC |
|  | g-Prevo-R | GGTCGGGTTGCAGACC |
| *Clostridium difficile* | Cd-lsu-F | GGGAGCTTCCCATACGGGTTG |
|  | Cd-lsu-R | TTGACTGCCTCAATGCTTGGGC |
| *Clostridium perfringens* | s-Clper-F | GGGGGTTTCAACACCTCC |
|  | ClPER-R | GCAAGGGATGTCAAGTGT |
| *Lactobacillus* | sg-Lgas-F | GATGCATAGCCGAGTTGAGAGACTGAT |
| (formerly *Lactobacillus gasseri* subgroup) | sg-Lgas-R | TAAAGGCCAGTTACTACCTCTATCC |
| *Lacticaseibacillus* | sg-Lcas-F | ACCGCATGGTTCTTGGC |
| (formerly *Lactobacillus casei* subgroup) | sg-Lcas-R | CCGACAACAGTTACTCTGCC |
| *Enterobacteriaceae* | En-lsu-3F | TGCCGTAACTTCGGGAGAAGGCA |
|  | En-lsu-3'R | TCAAGGACCAGTGTTCAGTGTC |
| *Enterococcus* | g-Encoc-F | ATCAGAGGGGGATAACACTT |
|  | g-Encoc-R | ACTCTCATCCTTGTTCTTCTC |
| *Streptococcus* | g-Str-F | AGCTTAGAAGCAGCTATTCATTC |
|  | g-Str-R | GGATACACCTTTCGGTCTCTC |
| *Staphylococcus* | g-Staph-F | TTTGGGCTACACACGTGCTACAATGGACAA |
|  | g-Staph-R | AACAACTTTATGGGATTTGCWTGA |
| *Pseudomonas* | PSD7F | CAAAACTACTGAGCTAGAGTACG |
|  | PSD7R | TAAGATCTCAAGGATCCCAACGGCT |

**Table S1: 16S and 23S rRNA gene-targeted primers used in this study**

**Table S2**

|  |  | Control |  | Day 1 | |  | Day 3 | |  | Day 7 | |  | Day 14 | |
| --- | --- | --- | --- | --- | --- | --- | --- | --- | --- | --- | --- | --- | --- | --- |
|  |  |  |  | Syngeneic | Allogeneic |  | Syngeneic | Allogeneic |  | Syngeneic | Allogeneic |  | Syngeneic | Allogeneic |
| Succinic acid |  | 1.57 ± 0.70 |  | 1.16 ± 0.34 | 1.08 ± 0.19 |  | 1.18 ± 0.47 | 0.71 ± 0.33 |  | 0.62 ± 0.23 | 0.92 ± 0.14 |  | 1.77 ± 0.73 | 0.84 ± 0.17 |
| Valeric acid |  | 0.4 |  | 1.26 ± 0.54 | 1.22 ± 0.17 |  | 1.51 ± 0.61 | 0.60 ± 0.18 |  | 1.52 ± 0.61 | 0.46 ± 0.12 |  | 1.83 ± 0.62 | 0.33 |
| Isovaleric acid |  | 0.4 |  | 0.67 ± 0.25 | 1.24 ± 0.20 |  | 0.4 | 1.05 ± 0.23 |  | 0.4 | 0.80 ± 0.28 |  | 0.4 | 0.67 ± 0.25 |

**Table S2: Time-series change in other fecal organic acids**

Values are mean ± SE (μmol/g of feces)
